# Case Report: An association of left ventricular outflow tract obstruction with 5p deletions

**DOI:** 10.3389/fgene.2024.1451746

**Published:** 2024-10-18

**Authors:** Kira Mascho, Svetlana A. Yatsenko, Cecilia W. Lo, Xinxiu Xu, Jennifer Johnson, Lindsey R. Helvaty, Stephanie Burns Wechsler, Chaya N. Murali, Seema R. Lalani, Vidu Garg, Jennelle C. Hodge, Kim L. McBride, Stephanie M. Ware, Jiuann-Huey Ivy Lin

**Affiliations:** ^1^ Division of Pediatric Critical Care, Rainbow Babies and Children’s Hospital, Cleveland, OH, United States; ^2^ University of Pittsburgh, Pittsburgh, PA, United States; ^3^ UPMC Children’s Hospital of Pittsburgh, Pittsburgh, PA, United States; ^4^ Department of Pediatrics, Indiana University School of Medicine, Indianapolis, IN, United States; ^5^ Division of Medical Genetics, Emory University School of Medicine, Atlanta, GA, United States; ^6^ Department of Molecular and Human Genetics, Baylor College of Medicine, Houston, TX, United States; ^7^ Center for Cardiovascular Research and Heart Center, Nationwide Children’s Hospital, Columbus, OH, United States; ^8^ Department of Medical Genetics, University of Calgary, Calgary, AB, Canada; ^9^ Department of Medical and Molecular Genetics, Indiana University School of Medicine, Indianapolis, IN, United States; ^10^ Department of Critical Care Medicine, University of Pittsburgh, Pittsburgh, PA, United States

**Keywords:** 5p deletion, congenital heart defect, genetic disorder, left ventricular outflow tract obstruction, copy number variant

## Abstract

**Introduction:**

5p deletion syndrome, also called Cri-du-chat syndrome 5p is a rare genetic syndrome with reports up to 36% of patients are associated with congenital heart defects. We investigated the association between left outflow tract obstruction and Cri-du-chat syndrome.

**Methods:**

A retrospective review of the abnormal microarray cases with congenital heart defects in Children’s Hospital of Pittsburgh and the Cytogenomics of Cardiovascular Malformations Consortium.

**Results:**

A retrospective review at nine pediatric centers identified 4 patients with 5p deletions and left outflow tract obstruction (LVOTO). Three of these patients had additional copy number variants. We present data suggesting an association of LVOTO with 5p deletion with high mortality in the presence of additional copy number variants.

**Conclusion:**

A rare combination of 5p deletion and left ventricular outflow obstruction was observed in the registry of copy number variants and congenital heart defects.

## Introduction

5p deletion syndrome or 5p minus syndrome, also called Cri-du-chat syndrome, is a rare genetic syndrome that was first described with the distinctive, high-pitched, cat-like cry in 1963 by Lejeunne et al. (Lejeune et al., 1963). In Frech, Cri-du-chat translates to “cry of the cat.” The most recognizable phenotypes are the characteristic shrill cry like the mewing of a cat, distinctive facial features, growth and developmental delay (Nguyen et al., 2015). However, there is a wide spectrum of features in the individuals with 5p deletion syndrome that may be attributed to the differences in their genotypes in whether terminal or interstitial 5p deletions occur at different breakpoints or the deleted genes in the 5p region (Nguyen et al., 2015). Cri-du-chat syndrome is a contiguous gene syndrome (Gersh et al., 1995). Studies by Overhauser et al. (1994) determined the deletion of 5p15.2 was correlated with facial dysmorphism and developmental delays and the deletion of 5p 15.3 was related to the characteristic cat-like cry (Gersh et al., 1995). Additional analysis by Zhang et al., in 2005 further localized the region of the cat-like cry to 5p 15.31, facial dysmorphism to 5p15.2 to 5p15.31, and speech delay to 5p15.32 to 5p 15.33 (5). Congenital heart defects (CHDs) are reported in approximately 15%–36% of 5p deletion syndrome, typically representing simple heart defects such as ventricular septal defect (VSD), atrial septal defect (ASD), patent ductus arteriosus (PDA), tetralogy of Fallot (TOF) or aortic stenosis (AS) (Hills et al., 2006; Mainardi et al., 2006; Nevado et al., 2021). Congenital heart defects are one of the most common causes of 5p deletion death (Nguyen et al., 2015). Left-sided lesions including left ventricular outflow tract obstruction (LVOTO) malformations have rarely been reported in 5p deletion syndrome. Nevado et al. (2021) observed up to 25% of additional copy number variants (CNVs) were noted in 5p deletion syndrome cohorts. Here we report two detailed case presentations of 5p deletion and LVOTO anomalies. Two more patients with 5p deletion in the presence of other CNVs were identified through a large multi-institution collaboration.

## Methods

A retrospective review was performed on abnormal chromosomal microarray (CMA) cases (abnormal CNVs were detected either by SNP microarray or array CGH) with CHDs from UPMC Children’s Hospital of Pittsburgh (CHP) and the Cytogenomics of Cardiovascular Malformations (CCVM) Consortium (Landis et al., 2023). The expression of related genes in the human heart tissues was obtained from the GTEx portal (https://gtexportal.org/home/singleCellOverviewPage). Enrichment of transcription factor targets was rendered using Metascape for genes within 5p deletions identified in a separate cohort of four complex CHD patients with LVOTO.

### Case description

#### Patient 1 (009–0116)

**FIGURE 1:**
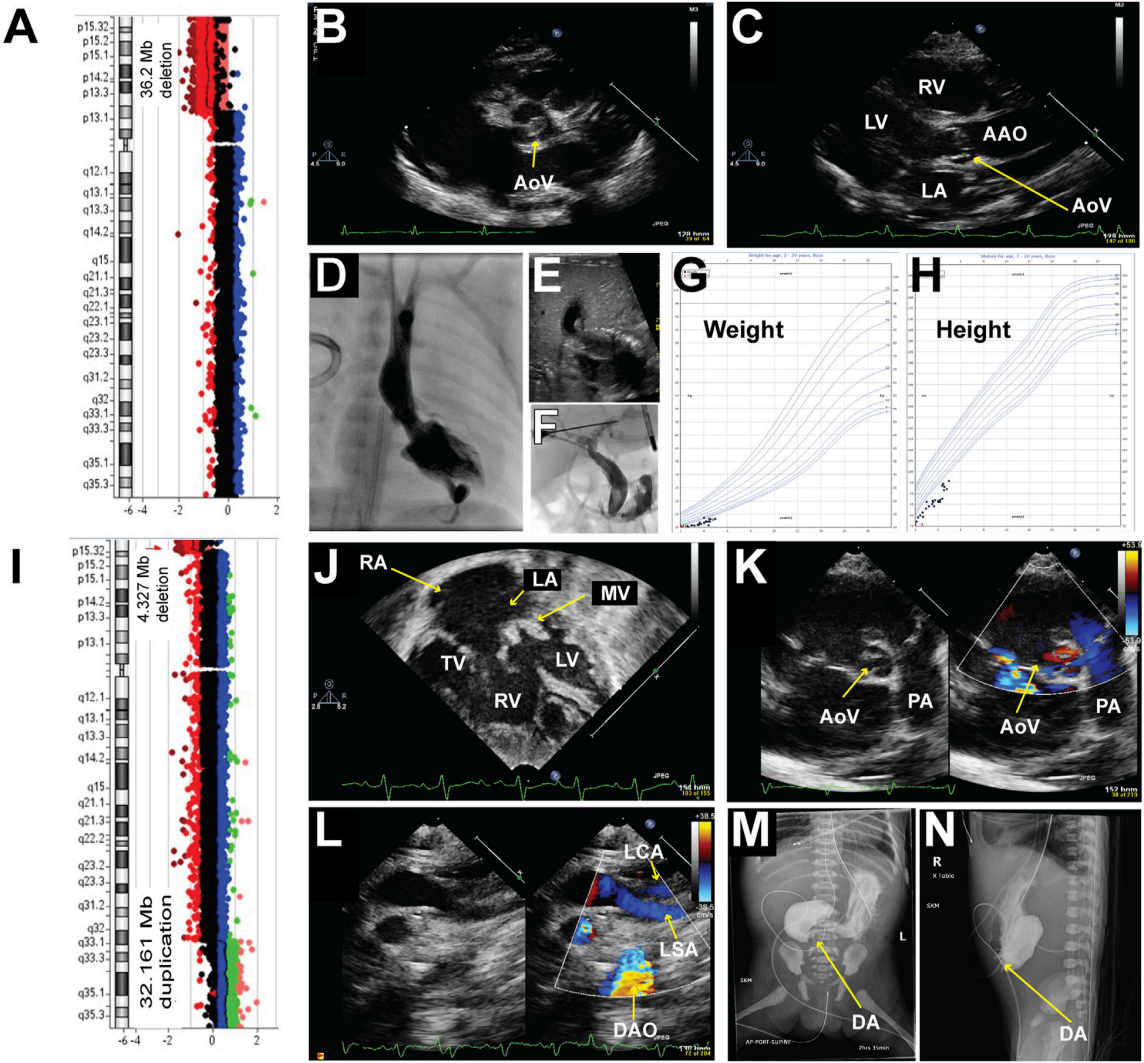
Chromosomal abnormalities and imaging from patients 1 and 2. Patient #1 **(A–H)** A Chromosomal microarray detected a 35.2 Mb deletion at 5p, specifically arr [GRCh37] 5p15.33p13.2 (22149_36232545) x1. **(B–C)**: Representative echocardiogram imaging demonstrating the patient’s dysplastic aortic valve and post-stenotic dilatation of the ascending aorta. **(D)** Left ventriculogram demonstrating an apex-forming left ventricle with a hypoplastic aortic valve. The aortic valve annulus measured 4.46 mm in the anterior-posterior projection with mild post-stenotic dilation of the ascending aorta. A posterior pseudoaneurysm was noted with a small track extending apically through the myocardium. **(E)** Representative abdominal ultrasound image demonstrated dilation of the left intrahepatic biliary system as well as the intrahepatic common bile duct with gall sludge within the intrahepatic common bile duct. **(F)** An injection of contrast from a micro-puncture needle into a left hepatic duct filled a mildly dilated segmental biliary branch and communication with the dilated common hepatic and biliary ducts. **(G)** Growth chart of weight. **(H)** Growth chart of height. Patient #2 **(I–N) (I)** Chromosomal microarray detected a 4.327 Mb deletion at 5p, specifically arr [GRCh37] 5p15.33 (22149_4349495) x1, and a 32.161 Mb duplication at 5q, specifically arr [GRCh37] 5q32q35.3 (148535314_180696806) x3. **(J–L)**: Representative echocardiogram imaging demonstrating a hypoplastic left ventricle, dysplastic and thickened mitral valve, large secundum atrial septal defect (ASD), large ventricular septal defect (VSD), and a bicuspid aortic valve with thickened leaflets. Color flow Doppler in image **(L)** showed a lack of continuity between the left subclavian artery and the descending aorta consistent with interrupted aortic arch type **(A)**. **(M–N)**: Upper GI with small bowel follow-through demonstrating opacification of the stomach and blind-ending proximal duodenum is consistent with duodenal atresia. AAO = Ascending Aorta, DAO = Descending Aorta, LA = Left Atrium, LV = Left Ventricle, LSA = Left Subclavian Artery, MV = Mitral Valve, PA = Pulmonary Artery, RA = Right Atrium, RV = Right Ventricle, TV = Tricuspid Valve.

A male was born at 36–5/7-week gestational age with prenatal concerns for aortic valve stenosis, aortic arch abnormality, and intrauterine growth restriction (IUGR). At birth, his weight was 2.078 kg (1.24 percentile), length was 44 cm (1.23 percentile), and head circumference was 31 cm (0.12 percentile). He demonstrated microcephaly, prominent supraorbital ridge, hypertelorism, down-slanting palpebral fissures, prominent nasal root, and retrognathia. Right single palmar crease, tapered digits, and fifth digit clinodactyly were noted in his upper extremities. Bilateral metatarsus adductus and bilateral sandal gap between his first and second toes were noted in his lower extremities. An echocardiogram showed a dysplastic aortic valve with aortic stenosis (Figures 1B, C) and arch hypoplasia. The patient underwent aortic valvuloplasty at 2 days of life which was complicated by a posterior left ventricle (LV) pseudoaneurysm (Figure 1D). After the procedure there continued to be a significant gradient across the aortic valve and aortic arch with the concern of arch obstruction, therefore, he underwent arch reconstruction and aortic valvuloplasty at 10 days of life. He was noted to have a bicuspid aortic valve oriented in the anterior and posterior axis. There was a fusion of the commissures anteriorly. The aortic valve looked dysplastic and thickened. In addition, there was a raphe at the level of the junction between the right and left coronary cusps. His postoperative course was complicated by left vocal cord hypomobility and feeding difficulties. Subsequently, he was diagnosed with obstructive jaundice and was treated with biliary drainage (Figures 1E, F) when he was 2 months old. The drain was removed about a month later with no further issues. An echocardiogram at 5 months of age demonstrated the evolution of LV pseudoaneurysm. Given his congenital heart anomalies and multiple dysmorphic features, chromosomal microarray testing was ordered, which revealed a large deletion at 5p13.33-p13.2, specifically arr [GRCh37] 5p15.33p13.2 (22149_36232545) x1 encompassing 36.3 Mb and at least 320 genes (Figure 1A), including the critical region associated with Cri-du-chat syndrome which was identified in most studies as the region between 5p15.3-5p15.2 (Gersh et al., 1995; Overhauser et al., 1994; Church et al., 1995; Wu et al., 2005) and a 5.5 Mb deletion of 5p15.33-p15.32 (arr 5p15.33p15.32 (22178–5539182) x1 in a 3-generation family with atypical Cri-du-chat syndrome (Elmakky et al., 2014). This deletion also contains a 5p15 terminal (Cri-du-chat syndrome) region with sufficient evidence for haploinsufficiency (Zhang et al., 2005; Mainardi et al., 2006). Although he had multiple complications during his initial neonatal hospitalization and delayed milestones, he has been gaining weight and thriving on his growth curve (Figures 1G, H). He is 5 years old and is the only documented survivor in this case series, with the largest chromosomal deletion in the 5p region and no concurrent reported other CNVs. Based on ACMG guidelines this deletion is classified as pathogenetic CNV.

#### Patient 2 (009–0119)

A 37–4/7-week gestational age female was prenatally diagnosed with Cri-du-chat syndrome, had a 4.327 Mb deletion at 5p, specifically arr [GRCh37] 5p15.33 (22149_4349495) x1, and a 32.161 Mb duplication on 5q, specifically arr [GRCh37] 5q32q35.3 (148535314_180696806) x3 (Figure 1I). This deletion also contains 5p15 terminal (Cri- du-chat syndrome) region with sufficient evidence for haploinsufficiency (Zhang et al., 2005; Mainardi et al., 2006). The duplication contains 5q35 recurrent (Sotos syndrome) region (includes *NSD1*) with sufficient evidence for triplosensitivity, which is associated with the Sotos phenotype (Rosenfeld et al., 2013). Prenatal ultrasound at around 20 weeks of gestational age demonstrated duodenal atresia and a decrease in LV function. She was born to a 26-year-old woman (gravida 7, para 1) with a history of six spontaneous abortions during prior pregnancies and a pericentric inversion- 46,XX,inv (Zhang et al., 2005) (p15.3q32). At birth, her weight was 1.665 kg (<0.01 percentile), length was 42.5 cm (<0.01 percentile), and head circumference was 30 cm (0.01 percentile). She was microcephalic, and her birth height and weight were below average. She was born without spontaneous respiratory effort, necessitating an emergent intubation in the delivery room. Her initial heart rate at birth was less than 60 bpm without spontaneous movement. Her Apgar scores were 1 at 1 min, 3 at 5 min, and 3 at 10 min. Multiple facial dysmorphisms, including hypertelorism, low-set ears, and micrognathia, were noted after delivery. An echocardiogram was obtained due to severe metabolic acidosis and a murmur which demonstrated a hypoplastic LV, severe mitral valve dysplasia, a large VSD, a large ASD secundum (Figure 1J), a dysplastic aortic valve (Figure 1K) with an interrupted aortic arch type A (Figure 1L) and a large PDA. The upper gastrointestinal series confirmed the diagnosis of duodenal atresia (Figures 1M, N). She required multiple inotropic agents with a maximum Wernovsky inotrope score of 20 after birth. In conjunction with her comorbidities and ongoing critical illness, surgical palliation was not offered to her. She passed away on the day of life 6. Based on ACMG guidelines, deletion and duplication are classified as pathogenetic CNV(14). Also, this result suggests a parental balance inversion origin, genetic counseling is recommended. We found that the patient’s mom carries a history of six spontaneous abortions during prior pregnancies and a pericentric inversion- 46, XX, inv (Zhang et al., 2005) (p15.3q32).

#### Additional patients

**FIGURE 2:**
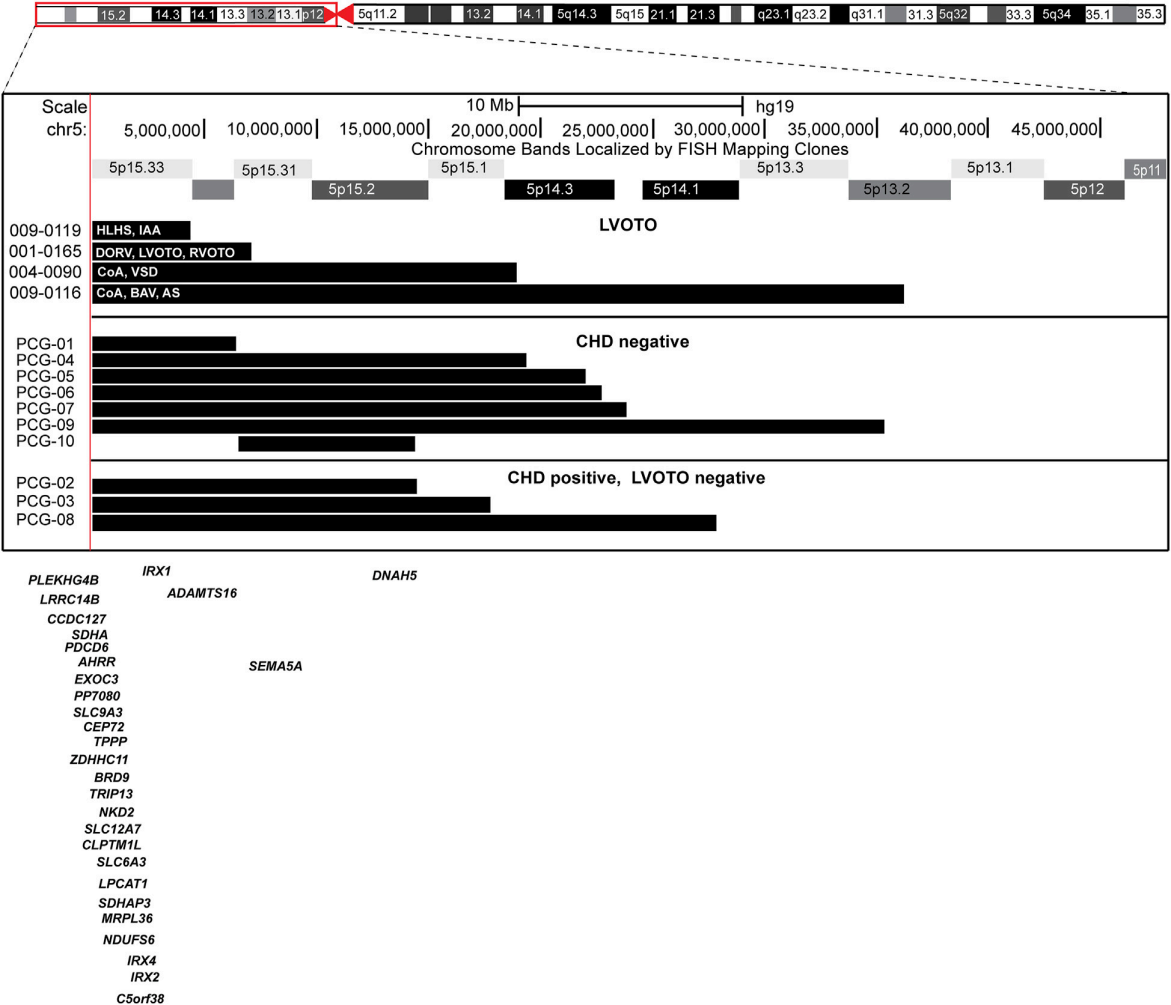
Ideogram of chromosome 5 with the expansion of the 5p region and location of the individual 5p deletions in each patient.

We identified five more patients with 5p deletion (Figure 2; Table 1; Supplementary Table S1) and LVOTO malformation from the CCVM Consortium (Hinton et al., 2015). This multi-site, cross-disciplinary collaboration has created a large database registry of patients with CHD who have had non-normal clinical CMA results. A total of 1,363 patients from nine pediatric centers across the United States were included in the study (Landis et al., 2023). There are 229 individuals with LVOTO in the CCVM Consortium. We excluded three patients who had small 5p deletion that is not consistent with the typical 5p deletion syndrome, with the size of deletion of 5p varying from 0.01 to 0.27 MB, which is less than the reported deletion size from 5 to 40 MB in Cri-du-chat syndrome and no genes in the deleted region that relate to heart development as well as not consistent with the typical 5p deletion syndrome (Simmons et al., 1995; Cerruti Mainardi, 2006) and these small deletions were not consistent with pathological CNV according to ACMG guidelines (Rosenfeld et al., 2013). These 3 patients also have additional CNVs that are known to cause CHDs (Supplementary Table S1). The major birth defects of these additional 2 patients we included are summarized in Table 1. In total, there are four patients with a deletion in regions associated with Cri-du-chat syndrome (5p15.2-5p15.3, patients 1–4) (Zhang et al., 2005; Mainardi et al., 2006) in this report, of whom three patients had additional CNVs. Patient 2 (009–0119) had a 5q duplication in the region containing *HAND1* and *Nkx2-5* (Supplementary Table S2). Patient 3 (001–0165) had a 7.1 Mb deletion arr [hg19] 5p15.33p15.31966648_7175604) x1, and an 8p duplication in the region containing *MYOM2*. Interstitial duplication of 8p23 was noted to be associated with CHDs but 8p trisomy has not been reported to be associated with LVOTO (Gug et al., 2020). Patient 3 has a complex CHD with a double outlet right ventricle (DORV) and both left and right ventricular outflow tract obstruction. Patient 4 (004–0090) had an 18.9 Mb deletion arr [GRCh37] 5p15.33p14.3 (22149_18927458) x1 and arr [GRCh37]5p14.3p11 (19049019_46115173) x3, the 18.0 Mb duplication and a complex rearrangement of chromosome 5. Of these 4 patients, 2 died before 12 months of age and one did not have updated clinical information.

**TABLE 1:** Patients characteristics.

Patient Number	ID	GA^e^ (weeks)	Birth Body weight (kg)	Sex	Vital status	Cardiac Defect	Other diagnoses	Genetic Findings	Interval size (Mb^j^)	5P minus syndro me
1	009- 0116	36-5/7	2.078	M^i^	Alive	Hypoplastic aortic arch, AS^a^	IUGR^g^, dys morphic facial features, distal extremity abnormalities	arr[GRCh37] 5p15.33p13.2(22149- 36232545x1)	5p deletion: 36.21	Yes
2	009- 0119	37-4/7	1.665	F^d^	Decea sed age 6 days	HLHS^f^, interrupted aortic arch, mitral valve dysplasia, large VSD^l^, ASDII^b^, PDA^k^	IUGR^g^, duodenal atresia, subdural hemorrhages respiratory failure	arr[GRCh37] 5p15.33(22149- 4349495)x1	5p deletion: 4.33	Yes
								arr[GRCh37] 5q32q35.3(148535314- 180696806)x3	5q duplication: 32.16	
3	001- 0165	Unknown	Unknown	M^i^	Unkno wn	Double outlet right ventricle (tetralogy of Fallot type); LVOTO^h^, muscular VSD^l^, ASDII^b^, aortic root dilatation	8p partial trisomy, other extracardiac abnormalities unknown	arr[hg18] 5p15.33p15.31(66,648- 7,175,604)x1	5p deletion: 7.11	Yes
								arr[hg18] 8p23.3p21.2(213- 26,130,535)x3	8p duplication: 26.13	
4	004- 0090	35	1.432	F^d^	Decea sed age 11 months	CoA^c^, perimembranous VSD^l^, ASDII^b^, bilateral superior vena cava	Dysmorphism tethered cord, metabolic acidosis, pulmonary insufficiency, congenital hypotonia, abdominal wall hernia, IUGR^g^	arr[GRCh37] 5p15.33p14.3(22,149- 18,927,458)x1	5p deletion: 18.91	Yes
								arr[GRCh37] 5p14.3p11(19,049,019- 46,115,173)x3	5p duplication: 27.07	

^a^AS: Aortic stenosis; ^b^ASDII: Atrial septal defect secundum; ^c^CoA: coarctation of the aorta; ^d^F: Female; ^e^GA, gestational age; ^f^HLHS: hypoplastic left heart syndrome; ^g^IUGR, intrauterine growth restriction; ^h^LVOTO, left ventricular outflow tract obstruction; ^i^M: male; ^j^Mb, megabases; ^k^PDA: Patent ductus arteriosus; ^l^VSD: Ventricular septal defect.

In summary, the 4 patients’ 5p deletions are classified as pathogenetic CNV based on ACMG guidelines (Riggs et al., 2020) with an incidence of 1.3% in this cohort (Landis et al., 2023). We reported the association of LVOTO and 5p deletion and observed an association of LVOTO and 5p deletion with high mortality in the presence of additional copy number variants. This also indicates an expansion of the cardiac abnormalities’ spectrum in the 5p deletion patients.

### No common critical region for LVOTO defects in 5p deletion patients

To identify the specific region associated with LVOTO in patients with 5p deletion, we compared 10 patients with 5p deletion at CHP to the four with LVOTO. Of these 10, seven were without CHD and 3 had non-LVOTO CHD (Figure 2). There is not a common region associated with 5p deletion and LVOTO in this case series. This could be due to incomplete penetrance or the etiology of LVOTO may be concurrent copy abnormalities other than 5p deletion.

## Discussion

Hills et al. reviewed a database of 98,000 congenital heart disease patients and identified twenty-one with Cri-du-chat syndrome (Hills et al., 2006). When characterized by the most hemodynamically significant lesion, twenty-one patients either had a VSD, PDA, Tetralogy of Fallot, or right ventricular outflow tract obstruction (Hills et al., 2006). The patients described in our case series all had septal defects, but uniquely all had more hemodynamically significant congenital anomalies causing LVOTO. 5p deletion syndrome was reported to be associated with CHDs in 18%–36% of patients including AS (7). When characterized by the most hemodynamically significant lesion, these patients either had a VSD, PDA, or TOF (7). The patients described in our case series had LVOTO malformation, three patients (patients 2–4) have additional CNVs. This could be due to incomplete penetrance. In addition, two of four patients pass away before 12 months of age with complex rearrangements at 5p/5q, complex critical congenital heart defects and intrauterine growth retardation, and low birth weight. Birth weight less than 1.5 kg with a critical congenital heart defect was known to be less likely to survive hospital discharge (Kim et al., 2021). Early observations of 5p deletion syndrome reported a 9.7% (32 of 341 individuals) mortality in childhood in 1978 with 90% of deaths within the first year (Niebuhr, 1978). The mortality rate decreased to 6.4% in the report in 2006 with 64% of deaths in the first year of life (Mainardi et al., 2006). Mainardi et al. also observed a higher mortality rate with unbalanced translocations that include a 5p deletion than the individuals with terminal deletions (18.5% vs. 4.8%) (Mainardi et al., 2006).

The etiology of CHD is multifactorial, and both epidemiologic studies and patient cohorts with chromosomal microarray testing have reported approximately 11%–18% of patients with CHD also have an identifiable syndromic genetic diagnosis (Helm et al., 2021), while 15%–21% of subjects with isolated CHD had LVOTO (Helm et al., 2021; Hoang et al., 2018). Genes known to be involved in cardiac expression are located on 5p (Supplementary Tables S3 and S4), including *DNAH5* (5p15.33, in patients 2, 4), NDUFS6 (5p15.33, in patients 1–4), IRX4 (5p15.33, in patient 1–4), and ADAMTS16 (5p15.32, in patient 2–4) (Pervolaraki et al., 2018). *ADAMTS16* p.H357Q variant is reported to be an inheritable human bicuspid aortic valve-related gene variant resulting from the fibronectin/ focal adhesion kinase (FAK) signal-mediated over-proliferation with extracellular matrix remodeling interruption (Lin et al., 2024). Patients 2 and 4 had a deletion in the *DNAH5* gene, a disease-causing variant related to laterality defects resulting from immotile cilia that lack dynein arms (Nöthe-Menchen et al., 2019). Patients 1–4 had a deletion in the region containing both *SDHA* and *NDUFS6*, complexes related to mitochondrial oxidative phosphorylation, which are highly expressed in the left ventricle (Pervolaraki et al., 2018). Intrinsic mitochondrial defects contribute to the different prognoses of single ventricle CHD, especially for HLHS (Xu et al., 2022). Patients 1–4 also had a deletion in the region containing *IRX4*, a homeobox gene with an expression in the ventricular myocardium (Bruneau et al., 2000). We observed the enrichment of five transcription factor targets (Subramanian et al., 2005) that are associated with heart development or defects (Supplementary Table S5) from the analysis of the affected genes within the 5p deletions in our patients. e.g., *DLX6* (Distal-Less Homeobox 6) directly regulates Basic helix-loop-helix transcription (bHLH) factor *HAND2*, which plays a crucial role for the development of the cardiac outflow tract (Holler et al., 2010). *FOXJ2*, a member of the Fork Head transcription factors family. Previous reports showed that there is a right/left heart difference in expression for *FOXJ2* (Philip-Couderc et al., 2008). *FOXJ2* also regulates Connexin-43 and E-Cadherin which may be associated with hypertrophic heart (Martin-de-Lara and Sanchez-Aparicio, 2008). These clues may explain how 5p deletion contributes to LVOTO.

The cases in this report suggest that the previous thought that 5p minus patients only have simple cardiac defects like ASD, VSD, and PDA, is not the case. There is a case report of a child with 5p minus and 20q duplication with Ebstein anomaly; the authors make a similar argument that this rare, complex congenital heart phenotype such as Ebstein has not ever been reported with 5p minus, though the 20q duplication may be part of the phenotype, too (Olivella et al., 2020). However, 5p deletion in the presence of other CNVs that are related to cardiac development may be associated with more complex hemodynamic significant cardiac defects. The correlation with LVOTO will be useful for clinical prognosis prediction on 5p deletion patients, as congenital heart defects are one of the most common causes of 5p deletion death (Nguyen et al., 2015). Significant hemodynamic complex congenital heart defects such as LVOTO increase the risk of mortality and morbidity. Further research is needed to elucidate an association between these genes and specific CHDs. In addition, further study of gene enhancers that may regulate gene expression from a distance or epigenetic regulation may provide additional insight into the critical regions for left heart formation and function on the short arm of chromosome 5.

## Conclusion

We present data suggesting an association of LVOTO and 5p deletion with high mortality in the presence of additional CNVs. Discovering new genotype-phenotype correlations for rare CHDs, including expanding the associations with known syndromes, is an important ongoing process requiring large patient cohorts with deep phenotyping such as that collected by the CCVM Consortium.

### Learning objectives

1. An association is suggested between LVOTO and 5p deletion, with high mortality occurring in patients with 5p deletion and other CNVs.
2. Expanding the phenotypic spectrum of known syndromes to include rare CHD requires multi-institution collaboration and expert-detailed heart phenotype evaluation.

## Data Availability

The original contributions presented in the study are included in the article/Supplementary Material, further inquiries can be directed to the corresponding author.

## Abbreviations

ASD, atrial septal defect; BAV, bicuspid aortic valve; CCVM, Cytogenomics of Cardiovascular Malformations Consortium; CHD, congenital heart defect; CMA, chromosomal microarray; CNV, copy number variants; CoA, coarctation of the aorta; HLHS, hypoplastic left heart syndrome; LVOTO, left outflow tract obstruction; VSD, ventricular septal defect.
