# Supplementary material for: Case Report: An association of left ventricular outflow tract obstruction with 5p deletions
Source: Front Genet. 2024 Oct 18;15:1451746. doi: 10.3389/fgene.2024.1451746 (PMC11527671; doi:10.3389/fgene.2024.1451746)
Supplement: Supplementary file 1 [file Table1.DOCX]

Supplemental Materials


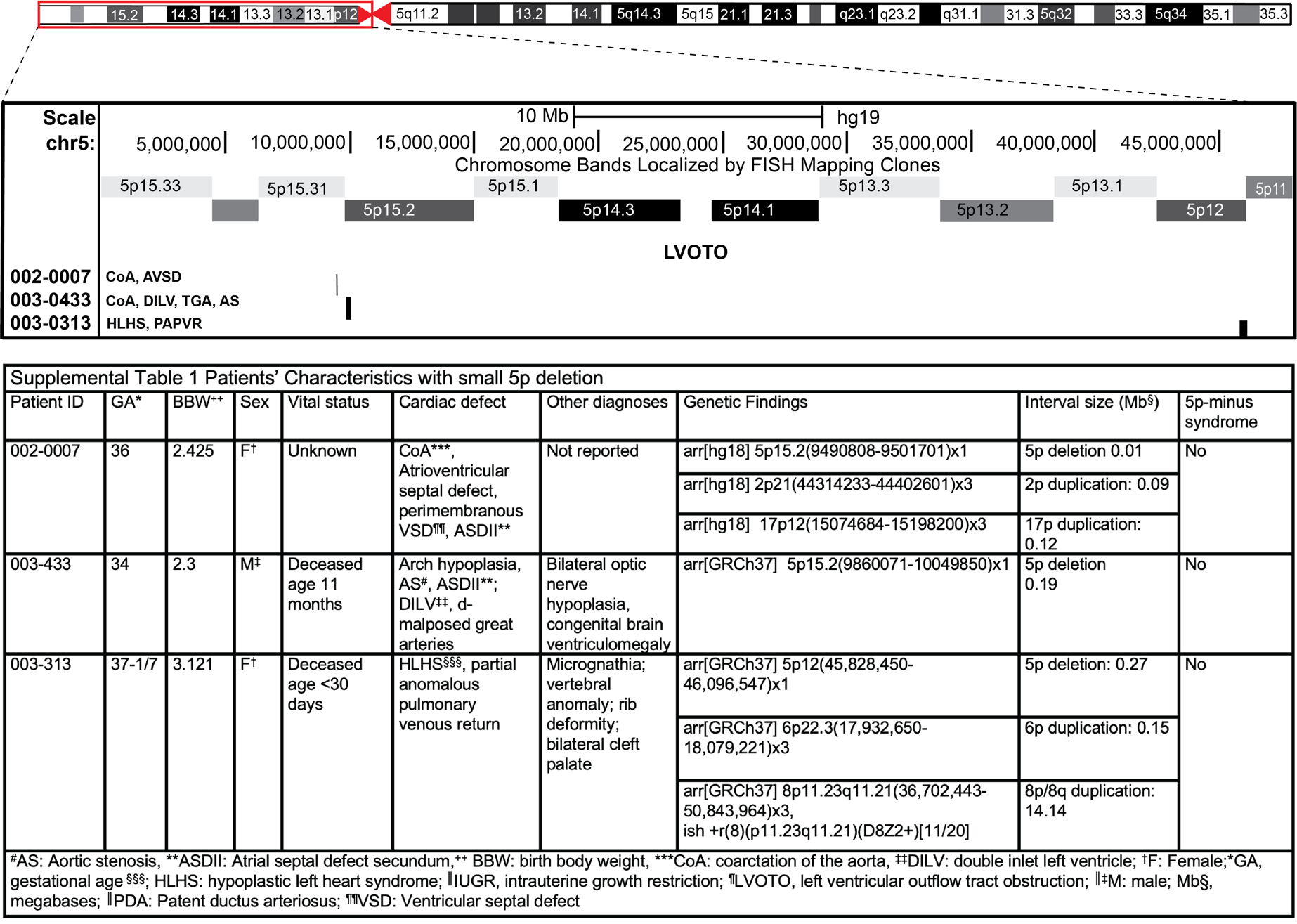


| Supplemental Table 2. Genes in Copy Number Alteration Other Than 5p Deletion in Patients | | |
| --- | --- | --- |
| Patient Number | Patient  ID | Affected Genes |
| 2 | 009-0119 | 7SK, AACSP1, ABLIM3, ADAM19, ADAMTS2, ADRA1B, AF157115, AFAP1L1, AGXT2L2, AK000953, AK001582, AK023159, AK055811, AK095057, AK098705, AK123543, AK123816, AK124680, AK126616, AK127224, AK309215, AK316321, AL049415, ANXA6, ARHGEF37, ARL10, ARSI, ATOX1, ATP10B, ATP6V0E1, AX746723, AX747080, AX747215, AX747764, AX747879, AX747985, AX748230, B4GALT7, BC005905, BC011998, BC034636, BC035392, BC036251, BC039346, BC040901, BC042064, BNIP1, BOD1, BTNL3, BTNL8, BTNL9, BX640700, BX648961, C1QTNF2, C5orf25, C5orf45, C5orf47, C5orf52, C5orf54, C5orf58, C5orf60, CAMK2A, CANX, CBY3, CCDC69, CCNG1, CCNJL, CD74, CDHR2, CDX1, CLINT1, CLK4, CLTB, CNOT6, CNOT8, COL23A1, CPEB4, CPLX2, REBRF, CSF1R, CSNK1A1, CYFIP2, DBN1, DCTN4, DDX41, DKFZp686M11215, DL490294, DOCK2, DOK3, DQ570121, DQ570256, DQ5760909, DQ578341, DQ579975, DQ589679, DQ596473, DQ596879, DQ601567, DQ601567, DQ658414, DRD1, DUSP1, EBF1, EFCAB9, EIF4E1B, ERGIC1, F12, FABP6, FAF2, FAM114A2, FAM153A, FAM153B, FAM153C, FAM193B, FAM196B, FAM71B, FAT2, FAXDC2, FBLL1, FBXW11, FGF18, FGFR4, FLJ16171, FLT4, FNDC6, FOXI1, G3BP1, GABRA1, GABRA6, GABRB2, GABRG2, GABRP, GALNT10, GEMIN5, GFPT2, GLRA1, GM2A, GMCL1P1, GNB2L1, GPRIN1, GPX3, GRIA1, GRK6, GRM6, GRPEL2, HAND1, HAVCR1, HAVCR2, HEIH, Hfb1, HI423497, HIGD2A, HK3, HMGXB3, HMMR, HMP19, HNRNPH1, HRH2, HRNRNPAB, IL12B, IL17B, IRGM, ITK, JA483376, JB074968, JB155089, KCNIP1, KCNMB1, KIAA1191, KIF4B, L26953, LARP1, LCP2, LINC00847, LMAN2, LOC100133331, LOC100268168, LOC100507387, LOC100507602, LOC100652758, LOC202181,LOC257358, LOC285593, LOC285626, LOC285627, LOC285629, LOC388312, LOC643201, LOC728554, LSM11, LTC4S, M55536, MAML1, MAPK9, MAT2B, MED7, Metazoa_SRP, MFAP3, MGAT1,MGAT4B, Mir_186, Mir_384, Mir_544, Mir_548, Mir_652, MIR103A1, MIR103B1, MIR1229, MIR143, MIR143HG, MIR146A, MIR218-2, MIR3141, MIR3142, MIR340, MIR378E, MIR3912, MIR4281, MIR4634, MIR4638, MIR585, MRPL22, MSX2,MXD3, MYOZ3, N4BP3, NDST1, NEURL1B, NHP2, NIPAL4, NKX2-5, NMUR2, NOP16, NPM1, NSD1, NUDCD2, ODZ2, OR2V1, OR2Y1, OR4F16, PANK3, PCYOX1L, PDEA6A, PDGF, PDGFRB, PDLIM7, PFN3, PPARGC1B, PPP1R2P3, PRELID1, PROP1, PRR7, PRR7-AS1, PTTG1, PWWP2A, RAB24, RANBP17, RARS, RASGEF1C, RBM22, RGS14, RMND5B, RNF130, RNF145, RNF44, RPL26L1, RPS14, RUFY1, SAP30L, SAP30L-AS1, SCGB3A1, SFXN1, SGCD, SH3PXD2B, SIMC1, SLC26A2, SLC34A1, SLC36A1, SLC36A2, SLC36A3, SLC6A7, SLIT3, SLU7, SMIM3, SNCB, SNORA40, SNORA74B,SNORD95, SNORD96A, SOX30, SPARC, SPDL1, SQSTM1, STC2, STK10, SYNPO, TBC1D9B, TCOF1, TENM2, THG1L, THOC3, TIGD6, TIMD4, TLX3, TMED9, TNIP1, TRIM41, TRIM52, TRIM7, TRNA, TRNA_Ala, TRNA_Leu, TRNA_Leu, TRNA_Lys, TRNA_Pro, TRNA_Pseudo, TRNA_Pseudo, TRNA_Thr, TRNA_Val, TSPAN17, TTC1, U4atac, U6, UBLCP1, UBTD2, UIMC1, UNC5A, WWC1, Y_RNA, ZFP2, ZFP62, ZNF300, ZNF300P1, ZNF346, ZNF354A, ZNF354B, ZNF354C, ZNF454, ZNF879 |
| 3 | 001-0165 | AK093412, AK128400, AK128880, ARHGEF10, AX747124, BC022082, BC037297, BC038783, BC045738, BC071667, C8orf42, CLN8, CR600473, CSMD1, DLGAP2, DQ584928, ERICH1, FBXO25, KBTBD11, KIAA0294, KIAA1890, MYOM2, OR4F21, RPL23A_20_869, ZNF596 |
| 4 | 004-0090 | ADAMTS12, AGXT2, AMACR, ANXA2R, BRIX1, C6, C7, C9, CAPSL, CARD6, C1QTNF3, CCDC152, CCL28, CHD6, CHD9, CDH10, CDH12, CHD18, C5orf17, C5orf22, C5orf34, CPLANE1, DAB2, DNAJC21, DQ596041, DQ591060, DROSHA, FBXO4, EGFLAM, FGF10, FYB1, GDNF, GDNF-AS1, GHR, GOLPH3, GUSBP1, HCN1, HMGCS1, IL7R, LIFR, LINC02109, LINC02120, LINC02241, LMBRD2, MIR4297, Mir_548, Mir_562, MROH2B, MRPS30, MTMR12, NIM1K, NNT, NADK2, NIPBL, NPR3, NUP155, OSMR, OXCT1, PAIP1, PDZD2, PLCXD3, PMCHL1, PRDM9, PRKAA1, PRLR, PTGER4, PURPL, RAD1, RAI14, RANBP3L, RICTOR, RPL37, RXFP3, SELENOP, SLC1A3, SLC45A2, SKP2, SPEF2, SUB1, TARS1, TMEM267, TTC23L, TTC33, UGT3A1, UGT3A2, WDR70, ZFR, ZNF131 |
|  |  | The genes with cardiac expression are highlighted in red |

| Supplemental Table 3: Genes in Copy Number Alteration of 5p Deletion in Patients 1-4 | | |
| --- | --- | --- |
| Patient Number | Patient ID | Affected Genes |
| 1 | 009-0116 | 5S_rRNA, ADAMTS12, ADAMTS16, ADCY2, AGXT2, AHRR, AK023178, AK094621, AK126225, AMACR, ANKH, ANKRD33B, ATPSCKMT, BASP1, BASP1-AS1, BRD9, BRIX1, C1QTNF3, C5orf17, C5orf22, C5orf38, C5orf49, CAPSL, CCDC127, CCT5, CDH10, CDH12, CDH18, CDH9, CEP72, CLPTM1L, CMBL CR749689, CTD-2297D10.2, CTD-3080P12.3, CTNND2, DAP, DNAH5, DNAJC21, DQ571461, DQ578105, DQ587763, DQ591060, DQ596041, DQ598099, DQ598168, DROSHA, EXOC3, EXOC3-AS1, FASTKD3, FBXL7, GOLPH3, GUSBP1, ICE1, IL7R, IRX1, IRX2, IRX4, LINC01018, LINC01019, LINC01020, LINC01194, LINC01511, LINC02103, LINC02111, LINC02112, LINC02114, LINC2120, LINC2145, LINC2199, LINC2223, LINC2226, LINC2239, LINC2241, LMBRD2, LOC100130744, LOC442132, LOC728613, LPCAT1, LRRC14B, LSP1P3, MARCHF6, MARCHF11, MED10, Mir_548, Mir_562, MIR4277, MIR4278, MIR4279, MIR4454, MIR4456, MIR4457, MIR4458, MIR4458HG, MIR4635, MIR4636, MIR4637, MIR580, MIR887, MRPL36, MTMR12, MTRR, MYO10, NDUFS6, NKD2, NPR3, OTULIN, OTULINL, PDCD6, PDCD6-AHRR, PDZD2, PLEKHG4B, PMCHL1, PP7080, PRDM9, PRLR, PURPL, RAD1, RAI14, RETREG1, RN7SKP133, RNU6-923P, ROPN1L, ROPN1L-AS1, RP11-19O2.2, RP11-43F13.3, RP11-310P5.1, RP11-804N13.1, RXFP3, SDHA, SDHAP3, SEMA5A, SKP2, SLC12A7, SLC45A2, SLC6A18, SLC6A19, SLC6A3, SLC9A3, SLC9A3-AS1, SNHG18, SNORD123, SPEF2, SRD5A1, SUB1, TARS1, TAS2R1, TENT4A, TERT, TPPP, TRIO, TRIP13, TRNA_Lys, TTC23L, UBE2QL1, UGT3A1, UGT3A2, Y_RNA, ZDHHC11, ZFR, ZNF622 |
| 2 | 009-0119 | AHRR, AK023178, AK126225, BRD9, C5orf38, CCDC127, CEP72, CLPTM1L, CR749689, CTD-3080P12.3, DQ598099, EXOC3-AS1, IRX1, IRX2, IRX4, LINC01019, LINC01511, LOC728613, LPCAT1, LRRC14B, MIR4277, MIR4456, MIR4457, MIR4635, MRPL36, NDUFS6, NKD2, PDCD6, PDCD-AHRR, PLEKHG4B, PP7080, RP11-310P5.1, RP11-43F13.3, SDHA, SDHAP3, SLC12A7, SLC6A18, SLC6A19, SLC6A3, SLC9A3, SLC9A3-AS1, TERT, TPPP, TRIP13, ZDHHC11 |
| 3 | 001-0165 | ADAMTS16, AHRR, AK094462, AK125283, AK126199, AL359941, ALG-2, BC032469, BC034612, BC034630, BC035019, BC043253, BC072677, BC150558, BRD9, C5orf38, C5orf49, CCDC127, CEP72, CLPTM1L, CR610608, CR621911, DKFZp434D0711, EXOC3, FLJ00157, FLJ25076, FLJ33360, hTERT, IRX1, IRX2, IRX4, KIAA0947, KIAA1234, KIAA2029, LOC116349, LOC340094, LOC389257, LOC728613, LPCAT1, MED10, MRPL36, NDUFS6, NKD2, NSUN2, PDCD6, PLEKHG4B, POLS, SAKI, SDHA, SLC12A7, SLC6A18, SLC6A3, SLC9A3, SRD5A1, TERT, TPPP, TRIP13, UNQ598, ZDHHC11 |
| 4 | 004-0090 | 5S_rRNA, ADAMTS16, ADCY2, AHRR, AK126225, ANKH, ANKRD33B, ATPSCKMT, BASP1, BASP1-AS1, BRD9, C5orf38, C5orf49, CCDC127, CCT5, CEP72, CLPTM1L, CMBL, CR749689, CTD-3080P12.3, CTD-2297D10.2, CTNND2, DAP, DNAH5, DQ598099, EXOC3, EXOC3-AS1, FASTKD3, FBXL7, ICE1, IRX1, IRX2, IRX4, LINC01018, LINC01019, LINC01020, LINC01194, LINC01511, LINC02111, LINC02112, LINC02114, LINC02145, LINC02199, LINC02223, LINC02226, LOC100130744, LOC442132, LOC728613, LPCAT1, LRRC14B, MARCHF6, MARCHF11, MED10, MIR4277, MIR4278, MIR4454, MIR4456, MIR4457, MIR4458, MIR4458HG, MIR4635, MIR4636, MIR4637, MIR887, MRPL36, MTRR, MYO10, NDUFS6, NKD2, NSUN2, OTULIN, OTULINL, PDCD6, PDCD6-AHRR, PLEKHG4B, PP7080, RN7SKP133, ROPN1L, ROPN1L-AS1, RP11-19O2.2, RP11-43F13.3, RP11-310P5.1, SDHA, SDHAP3, SEMA5A, SLC6A18, SLC6A3, SLC9A3, SLC9A3-AS1, SLC12A7, SNHG18, SNORD123, SRD5A1, TAS2R1, TERT, TPPP, TRIO, TRIP13, UBE2QL1, ZDHHC11, ZNF622 |
| The genes with cardiac expression are highlighted in red | | |

| Supplemental Table 4: Genes with cardiac expression in the chromosome 5p region | | | | | |
| --- | --- | --- | --- | --- | --- |
| Name | Gene | GTEx-Aorta | GTEx-Coronary | GTEx-Atrial Appendage | GTEx-Left Ventricle |
| ENSG00000073578.16 | SDHA | 93.1138 | 81.7462 | 206.773 | 301.984 |
| ENSG00000145494.11 | NDUFS6 | 58.4221 | 57.8943 | 78.9863 | 98.6259 |
| ENSG00000113504.20 | SLC12A7 | 19.0476 | 25.1314 | 33.7738 | 49.6748 |
| ENSG00000171421.12 | MRPL36 | 23.0724 | 24.5664 | 15.3627 | 18.0231 |
| ENSG00000049656.13 | CLPTM1L | 75.4104 | 70.3806 | 24.7702 | 16.0223 |
| ENSG00000185028.3 | LRRC14B | 0.0337074 | 0.0203147 | 11.5102 | 14.9192 |
| ENSG00000180104.15 | EXOC3 | 39.3756 | 36.0228 | 15.1896 | 12.1526 |
| ENSG00000249915.7 | PDCD6 | 45.8074 | 44.1837 | 14.9644 | 11.0359 |
| ENSG00000113430.9 | IRX4 | 0.0331038 | 0.0297951 | 0.0320179 | 8.25049 |
| ENSG00000028310.17 | BRD9 | 22.1889 | 21.6223 | 10.9659 | 7.80987 |
| ENSG00000171368.11 | TPPP | 14.094 | 10.7904 | 9.29339 | 6.51766 |
| ENSG00000153395.9 | LPCAT1 | 33.2939 | 28.0071 | 9.2918 | 4.91021 |
| ENSG00000164366.3 | CCDC127 | 6.14241 | 6.14136 | 4.31331 | 3.6488 |
| ENSG00000112902.11 | SEMA5A | 7.03307 | 7.09562 | 4.10806 | 3.29026 |
| ENSG00000185986.11 | SDHAP3 | 7.52455 | 7.28241 | 5.14907 | 3.03207 |
| ENSG00000188242.4 | PP7080 | 7.1906 | 8.3652 | 3.68208 | 2.84129 |
| ENSG00000145506.13 | NKD2 | 0.868825 | 2.16374 | 3.83755 | 2.18556 |
| ENSG00000188818.12 | ZDHHC11 | 1.86648 | 1.53582 | 3.95556 | 2.01323 |
| ENSG00000112877.7 | CEP72 | 1.67303 | 1.82461 | 0.864719 | 0.470354 |
| ENSG00000186493.11 | C5orf38 | 1.20144 | 2.30178 | 0.154621 | 0.460707 |
| ENSG00000170561.12 | IRX2 | 0.747252 | 1.28644 | 0.161886 | 0.427535 |
| ENSG00000066230.10 | SLC9A3 | 1.10945 | 1.5623 | 0.697472 | 0.235768 |
| ENSG00000170549.3 | IRX1 | 3.27814 | 0.127733 | 0.053197 | 0.224754 |
| ENSG00000071539.13 | TRIP13 | 0.823004 | 0.998673 | 0.295267 | 0.187056 |
| ENSG00000063438.16 | AHRR | 0.671068 | 0.693808 | 0.245242 | 0.12574 |
| ENSG00000153404.14 | PLEKHG4B | 0.0306904 | 0.0231053 | 0.0323986 | 0.0423593 |
| ENSG00000142319.17 | SLC6A3 | 0.171918 | 0.20794 | 0.0674917 | 0.0220713 |

| Supplemental Table 5. Transcription factor targets associated with heart development or defects | | |
| --- | --- | --- |
| Transcription Factor | Function | Ref |
| *DLX6* | Basic helix-loop-helix transcription (bHLH) factor Hand2 is the direct transcriptional target of Dlx5 and Dlx6, which plays a crucial role in the development of the cardiac outflow tract. | Barron, *et al.*(1)  Holler, *et al.*(2) |
| *FOXJ2* | FoxJ2, a member of the Fork Head transcription factors family. There is a right/left heart difference in expression for FoxJ2. FoxJ2-regulated Connexin-43 and E-Cadherin. FoxJ2 overexpression is associated with the hypertrophic heart. | Philip-Couderc, *et al.*.(3)  Martin-de-Lara,*et al.*(4) |
| *CDP* | Role of the multifunctional CDP/Cut/Cux homeodomain transcription factor in regulating differentiation, cell growth, and development. | Nepveu, *et al.*(5) |
| *IRF1* | Interferon Regulatory Factor 1 Is Required for Cardiac Remodeling in Response to Pressure Overload. | Jiang, *et al.*(6) |
| *ATF5* | Cardioprotection triggered by mitochondrial unfolded protein response is dependent on activating transcription factor 5 (ATF5). | Wang, *et al.*(7) |

Reference

1. Barron F, Woods C, Kuhn K, Bishop J, Howard MJ, Clouthier DE. Downregulation of Dlx5 and Dlx6 expression by Hand2 is essential for initiation of tongue morphogenesis. Development. 2011;138(11):2249-59.

2. Holler KL, Hendershot TJ, Troy SE, Vincentz JW, Firulli AB, Howard MJ. Targeted deletion of Hand2 in cardiac neural crest-derived cells influences cardiac gene expression and outflow tract development. Dev Biol. 2010;341(1):291-304.

3. Philip-Couderc P, Tavares NI, Roatti A, Lerch R, Montessuit C, Baertschi AJ. Forkhead transcription factors coordinate expression of myocardial KATP channel subunits and energy metabolism. Circ Res. 2008;102(2):e20-35.

4. Martin-de-Lara F, Sanchez-Aparicio P, Arias de la Fuente C, Rey-Campos J. Biological effects of FoxJ2 over-expression. Transgenic Res. 2008;17(6):1131-41.

5. Nepveu A. Role of the multifunctional CDP/Cut/Cux homeodomain transcription factor in regulating differentiation, cell growth and development. Gene. 2001;270(1-2):1-15.

6. Jiang DS, Li L, Huang L, Gong J, Xia H, Liu X, et al. Interferon regulatory factor 1 is required for cardiac remodeling in response to pressure overload. Hypertension. 2014;64(1):77-86.

7. Wang YT, Lim Y, McCall MN, Huang KT, Haynes CM, Nehrke K, et al. Cardioprotection by the mitochondrial unfolded protein response requires ATF5. Am J Physiol Heart Circ Physiol. 2019;317(2):H472-H8.
